# Gene-environment interaction in the pathophysiology of type 1 diabetes

**DOI:** 10.3389/fendo.2024.1335435

**Published:** 2024-01-26

**Authors:** Rahul Mittal, Nathanael Camick, Joana R. N. Lemos, Khemraj Hirani

**Affiliations:** ^1^Diabetes Research Institute, University of Miami Miller School of Medicine, Miami, FL, United States; ^2^Herbert Wertheim College of Medicine, Florida International University, Miami, FL, United States

**Keywords:** type 1 diabetes, genetics, gene-environment interaction, epigenetics, viral infections, pesticide exposure, pathological mechanisms

## Abstract

Type 1 diabetes (T1D) is a complex metabolic autoimmune disorder that affects millions of individuals worldwide and often leads to significant comorbidities. However, the precise trigger of autoimmunity and disease onset remain incompletely elucidated. This integrative perspective article synthesizes the cumulative role of gene-environment interaction in the pathophysiology of T1D. Genetics plays a significant role in T1D susceptibility, particularly at the major histocompatibility complex (MHC) locus and cathepsin H (CTSH) locus. In addition to genetics, environmental factors such as viral infections, pesticide exposure, and changes in the gut microbiome have been associated with the development of T1D. Alterations in the gut microbiome impact mucosal integrity and immune tolerance, increasing gut permeability through molecular mimicry and modulation of the gut immune system, thereby increasing the risk of T1D potentially through the induction of autoimmunity. HLA class II haplotypes with known effects on T1D incidence may directly correlate to changes in the gut microbiome, but precisely how the genes influence changes in the gut microbiome, and how these changes provoke T1D, requires further investigations. These gene-environment interactions are hypothesized to increase susceptibility to T1D through epigenetic changes such as DNA methylation and histone modification, which in turn modify gene expression. There is a need to determine the efficacy of new interventions that target these epigenetic modifications such as “epidrugs”, which will provide novel avenues for the effective management of T1D leading to improved quality of life of affected individuals and their families/caregivers.

## 1 Introduction

Type 1 diabetes (T1D) is a complex metabolic disorder characterized by the destruction of pancreatic β-cells due to autoimmunity leading to insulin deficiency and consequent hyperglycemia (1, 2). T1D is associated with a significant disease burden and its prevalence is increasing gradually (3). In 2021, there were an estimated 8.4 million people worldwide living with T1D (4). The prevalence of T1D has been reported to increase by 0.34% every year (4–6). By the year 2040, it is projected that the worldwide prevalence of T1D will potentially reach up to 17.4 million individuals (4–6). This represents a more than twofold increase within a span of 19 years (4). Furthermore, T1D has been associated with serious long-term complications, shortened life expectancy, and reduced quality of life (1, 2). In addition, T1D is a substantial economic burden on the healthcare system. In 2020, the lifetime economic burden of 1,630,317 patients with T1D in the United States was found to be $813 billion higher than an equal number of patients without T1D (7). In 2022 alone, the total estimated cost of diagnosed diabetes mellitus in the U.S. was $412.9 billion, including $306.6 billion in direct medical costs and $106.3 billion in indirect costs attributable to diabetes (8). The high disease burden and substantial healthcare costs associated with T1D underscore the urgent necessity to understand the precise molecular mechanisms underlying its pathophysiology, with the aim of developing effective prevention strategies, or ultimately cure for this disease.

Despite advances in the field of T1D, the precise trigger of autoimmunity and disease onset remain incompletely elucidated. A better understanding of the underlying pathophysiology will help in the identification of potential biomarkers and risk factors associated with T1D. This information will lead to the early detection of T1D and the development of preventive interventions to delay or even prevent its onset.

Genetics plays a crucial role in the pathophysiology of T1D (9–16). Individuals with a family history of the disease are at a higher risk, highlighting the hereditary nature of T1D. The primary genetic association is with specific human leukocyte antigen (HLA) genes, particularly those within the HLA-DR and HLA-DQ loci (15). Besides HLA, other genes such as cathepsin H (CTSH), *INS*, *GLIS3*, *CCR5*, and *BAD* have been implicated in predisposition to T1D (9–15). While genetic susceptibility has long been recognized as a key factor in T1D development, it is increasingly evident that environmental influences can play a pivotal role in shaping disease risk (17). Environmental factors such as viral infections and pesticide exposure have been shown to increase susceptibility to T1D (18–22). Although genetics and environmental factors individually have been associated with T1D, limited information is available regarding their cumulative contribution in the disease process. The interplay between genetic predisposition and environmental triggers is a dynamic and complex process, which may contribute to the heterogeneous nature of T1D.

This perspective article discusses the cumulative role of gene-environment interaction in the pathophysiology of T1D. We also discussed the potential molecular mechanisms through which this gene-environment interplay can trigger autoimmunity and predisposition to T1D. By synthesizing the latest research findings, we aim to elucidate the intricate mechanisms through which genetics and the environment converge to impact the risk and onset of T1D, ultimately paving the way for more targeted preventive and therapeutic strategies.

## 2 Genetic etiology of T1D

Genetics plays a significant role in T1D susceptibility, particularly at the major histocompatibility complex (MHC) locus, in addition to 59 other susceptibility loci (9–15, 23–30) (**Table 1**). These T1D risk variants are frequently found in regions that control gene activity across various cell types, including those within the exocrine pancreas (13).

**Table 1:** A summary of genes associated with the development of type 1 diabetes (T1D).

Gene	SNP	Function	Reference
*HLA Class II*	rs6927022 rs2157051 rs9275184 rs7744001	Antigen presenting complex for recognition by CD4+ T-cells	(31)
*CTLA4*	rs11571316 rs3087243	Protein receptor that downregulates immune reaction	(12, 32)
*CCR5*	rs113010081	Impacts immune cell function	(9)
*TLR7/8*	rs5979785	Receptor important for pathogen recognition and immune response activation	(33)
*AFF3*	rs9653442	Activates transcription, involved in oncogenesis and lymphoid development	(26)
*INS*	rs7111341	Insulin production; decreases blood glucose concentration	(27)
*GLIS3*	rs7020673 rs10758593	Participates in β-cell generation and insulin gene expression	(28)
*BAD*	rs694739	Initiates apoptosis and promoting cell death	(34)
*IL7R*	rs11954020	Involved in binding to antigens, production of immunoglobulins, and executing cell-mediated cytotoxic functions.	(9)
*IL10*	rs3024505	Anti-inflammatory cytokine	(32)
*IL27*	rs151234	Cytokine that regulates helper T-cell development and suppresses T-cell proliferation	(9)
*WFS1*	rs1046322	Mitigates endoplasmic reticulum stress in β-cells and allocortex of brain	(35, 36)
*CTSB*	rs1296023	Lysosomal enzyme necessary for protein degradation	(34)
*CTSH*	rs3825932	Lysosomal enzyme necessary for protein degradation	(32)
*GPX7*		Proliferation and apoptosis of pancreatic islet beta cells	(37)
*GSTT1*		Proliferation and apoptosis of pancreatic islet beta cells	(37)
*SNX19*		Proliferation and apoptosis of pancreatic islet beta cells	(37)

### 2.1 Human leukocyte antigen

There is an increased risk of developing T1D in individuals having mutations in the human leukocyte antigen (HLA) class II genes on chromosome 6, which contributes about 50% of the lifetime risk of this disease (15, 38). In particular, 90% of children with T1D possess either the DR4-DQ8 (DQA1*03:01 – DQB1*03:02) or the DR3-DQ2 (DQA1*05:01 – DQB1*02:01) haplotype. The combination of these two haplotypes in an individual’s genotype represents the highest risk factor for developing the disease (12). The relationship between *HLA* gene variants and T1D risk is a focus of extensive research. These genetic associations not only help in understanding the pathophysiology of T1D but also have implications for disease prediction and prevention strategies. For instance, HLA typing is used in risk stratification and in identifying individuals who may benefit from early interventions in T1D prevention trials (39).

### 2.2 Cathepsin H

Besides HLA, other gene loci have also been implicated in the development of T1D, namely the susceptibility locus of cathepsin H (CTSH). Genome-Wide Association Studies (GWAS) have associated CTSH with increased risk of developing T1D (40). A study determined the potential pathogenic mechanisms of the *CTSH* gene in T1D using integrated data from quantitative trait locus (eQTL) with GWAS (41). A marked overexpression of the *CTSH* gene in acinar cells was observed in pancreas from T1D patients compared to control group using single cell RNA sequencing (scRNA). Furthermore, utilizing single-cell weighted gene co-expression network analysis (WGCNA), a set of genes co-expressed with *CTSH* were identified that have a strong positive correlation with T1D. Based on functional enrichment analysis, it was hypothesized that the *CTSH* gene within the exocrine pancreas amplifies the antiviral response. This amplification leads to an increased expression of pro-inflammatory cytokines and the creation of an inflammatory microenvironment. Such a process is likely to cause injury to β cells, ultimately contributing to the development of T1D. Another study observed that the incidence of T1D was found to correlate with high CTSH expression, which itself is modified by other environmental factors such as epigenetics and post-translational modifications (42). Taken together, these studies highlight the role of *CTSH* in increased susceptibility of developing T1D.

### 2.3 Other genes

Other candidate genes such as *INS*, *GLIS3*, *CCR5*, *BAD*, *GPX7*, *GSTT1*, and *SNX19* have been shown to increase susceptibility to T1D (9–15, 23–30). Some of these genes directly affect the proliferation and apoptosis of pancreatic β-cells. A comprehensive list of genes associated with increased predisposition to T1D along with their function has been summarized in **Table 1**.

Although genetics have been found to play an integral role in the pathophysiology of T1D, recent studies have shown that development of T1D is multifactorial. Studies with identical twins have shown that if one twin develops T1D, the other twin may not show any susceptibility to the disease, suggesting that genetic factors alone cannot completely explain the development of T1D.

## 3 Environmental factors in the pathophysiology of T1D

Besides genetic etiology, environmental factors such as viral infections, pesticide exposure, lifestyle and dietary factors as well as vitamin D deficiency have all been individually associated with the development of T1D (17–19) (**Supplementary Table 1**).

### 3.1 Viral infections

**Figure 1:**
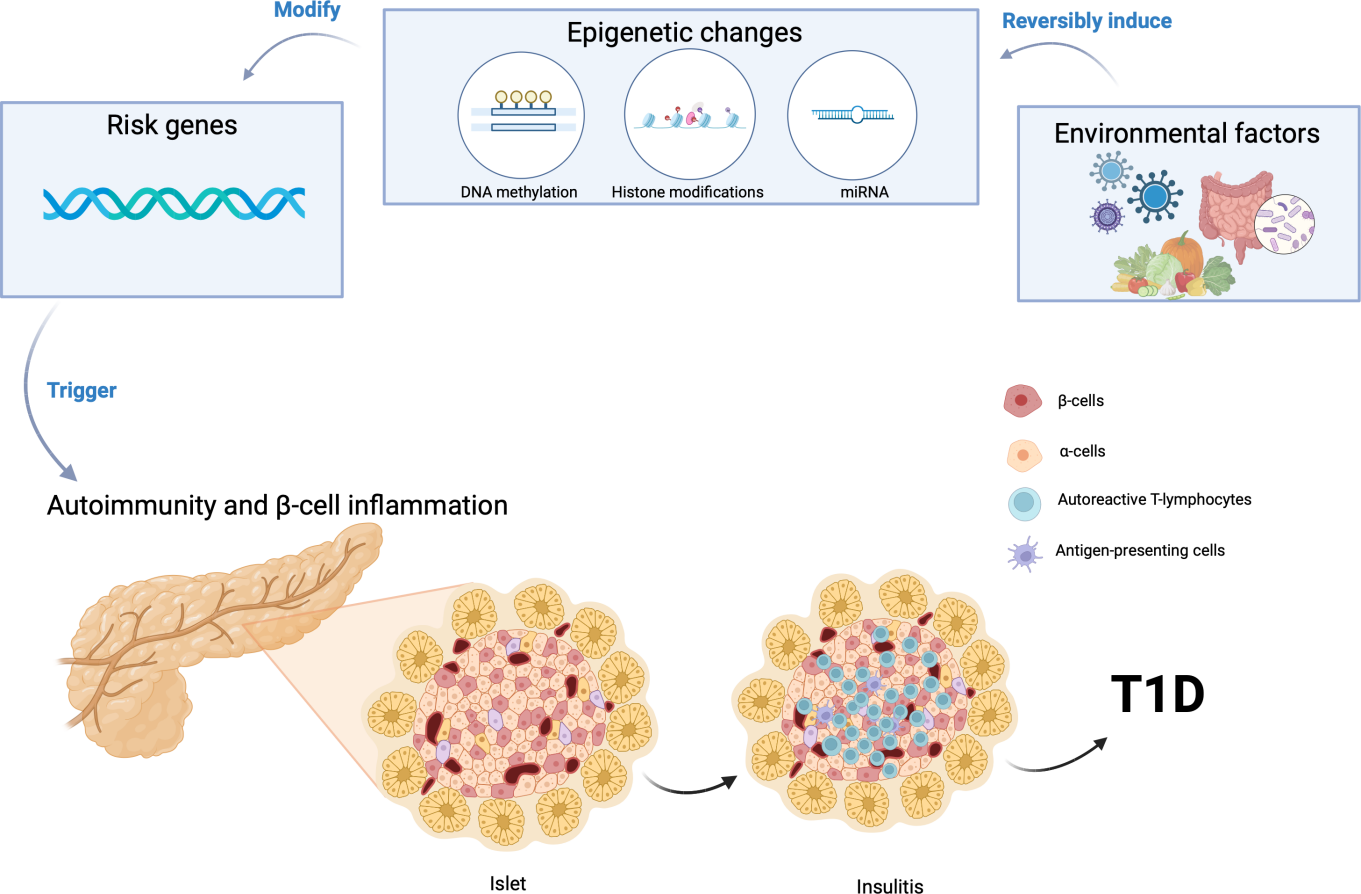
Gene-environment interaction can induce epigenetic modifications, initiating the autoimmune destruction of pancreatic β cells and consequently triggering the onset of Type 1 Diabetes (T1D). Created using BioRender.com.

Autoimmunity triggered by viral infections can play an important role in the etiology of T1D (**Figure 1**). Enteroviruses have been implicated at multiple levels in the etiopathogenesis of T1D, from infecting pancreatic β-cells to inducing autoimmunity against them (20). Cocksackie B viruses have been most frequently associated with the incidence of T1D (21, 22, 43). At the onset of disease in individuals with T1D, enterovirus proteins have been found in the pancreas (44). Since pancreatic β-cells also express several receptors used by enteroviruses to enter cells, various enterovirus species have been shown to infect and negatively impact the function of pancreatic β-cells (44). These viral infections trigger the production of interferons (IFN), which promote gene transcription; this IFN-stimulated gene expression has been shown in newly diagnosed T1D patients (44). This gene transcription has also been associated with the subsequent appearance of autoantibodies against pancreatic β-cells (44). As children with rapid onset T1D were found in the TEDDY study to be absent of viremia, this suggests that infections could induce autoimmunity progressively over time rather than acutely (45).

Furthermore, some viruses such as enteroviruses share structural similarities with pancreatic beta cell antigens. This resemblance may lead to a phenomenon known as molecular mimicry, where the immune system, activated to fight the virus, mistakenly attacks the body’s own cells, including insulin-producing beta cells and initiation of T1D (17).

### 3.2 Pesticide exposure

Pesticide exposure has been implicated in the development of T1D. Pesticides are chemicals designed to control pests and are widely used in agriculture, but their potential impact on human health has raised concerns. While research in this area is ongoing and findings are not conclusive, studies have explored the association between pesticide exposure and T1D (46). Epidemiological studies have suggested a possible link between pesticide exposure and T1D. Pesticide exposure has been associated with the incidence of T1D and prediabetes, termed abnormal glucose regulation, even at low concentrations (47). The causal relationship between pesticide exposure and abnormal glucose regulation differed between men and women, as a U-shaped dose-response relationship was more clearly demonstrated in men (47).

It has been hypothesized that pesticides may trigger or accelerate the autoimmune response that leads to beta-cell destruction in the pancreas. The mechanisms underlying this potential association are not fully understood but may involve the disruption of immune function or the induction of oxidative stress.

### 3.3 Other factors

#### 3.3.1 Mode of delivery and antibiotic use

Studies have suggested a correlation between antibiotic use and increased predisposition to T1D (48–50). The use of broad-spectrum antibiotics during the first two years of life has been associated with an increased risk of developing T1D depending on the mode of delivery (51). Intriguingly, the association of broad-spectrum antibiotics with T1D was only observed in children delivered through cesarean section but not in vaginally delivered babies (51). However, other studies do not observe any correlation between antibiotic use and T1D (52, 53). Further studies are warranted to decipher the effect of mode of delivery and antibiotic use in the development of T1D.

#### 3.3.2 Lifestyle and dietary factors

The influence of lifestyle and dietary factors on the development of Type 1 Diabetes (T1D) has been a subject of extensive research, revealing various associations and potential mechanisms (50). While the exact mechanisms are still being explored, it is evident that dietary habits leading to changes in gut microbiota composition may play a significant role in the development of T1D.

#### 3.3.3 Vitamin D deficiency

Low levels of Vitamin D have been associated with the development of T1D (54–57). This association is thought to be due to Vitamin D’s potential role in modulating the immune system, possibly impacting the autoimmune processes involved in T1D. However, other studies have observed no correlation between low levels of Vitamin D and a higher risk of T1D (58, 59). Additionally, the question of whether Vitamin D supplementation can reduce the risk of T1D remains under investigation, with mixed results from various studies (60). Further studies are warranted to elucidate the precise role of Vitamin D in T1D.

## 4 Gene-environment interaction and T1D

Despite the individual roles of genetic susceptibility and environmental risk factors, it is still unknown what triggers pancreatic β cell destruction and development of T1D in some patients. There is an emerging hypothesis that the interaction of environmental factors with genetic predisposition plays a crucial role in the pathophysiology of T1D (**Figure 1**). Environmental factors may exaggerate the effect of gene variants inducing autoimmunity and leading to the clinical manifestations of T1D.

Epigenetic modulators have emerged as pivotal regulators of gene expression and cellular phenotype, operating in conjunction with environmental factors (37, 61–66). Epigenetics is regarded as one of the primary molecular mechanisms by which gene-environment interactions may increase susceptibility to T1D (61, 62, 67). Epigenetic mechanisms such as DNA methylation alterations have been a focus of investigation, with findings indicating anomalous patterns in genes linked to immune function and insulin regulation in T1D individuals (68–71). Furthermore, histone modifications have shown their influence on immune response gene dysregulation in the context of T1D (72). The role of microRNAs, another facet of epigenetics, has also been underscored, particularly in controlling immune and inflammatory responses in T1D (61, 73–76). Epigenetic alterations associated with T1D risk not only hold implications for biomarker discovery but also open doors to precision medicine strategies in T1D diagnosis, risk assessment, and therapeutic intervention.

The other possible mechanism through which the gene-environment interaction can influence the onset of T1D is through alterations in the gut microbiome, which can impact mucosal integrity and immune tolerance (**Figure 1**) (77–81). This has been shown to increase the risk of T1D by increasing gut permeability through molecular mimicry and modulation of the gut immune system (82). Individuals with T1D and those at risk to develop T1D have exhibited an increase of *Bacteroides* and *Bifidobacterium* spp and a decrease of *Lactococcus* spp in their gut microbiome compared to healthy controls (82). Recent advancements in genetic technologies and gut microbiome determination techniques such as multi-omics signatures have allowed us to determine differences in the gut microbiome between patients with T1D and healthy controls at the functional level (83). In addition to the differences in *Bacteroides*, *Bifidobacterium*, and *Lactococcus* spp found in the gut microbiome, taxonomic analyses of the gut microbiota identified 51 species that differed in absolute abundance between T1D and healthy controls, with T1D patients showing increases in 17 species and decreases in 34 species (83). HLA class II haplotypes with known effects on T1D incidence may directly correlate to changes in the gut microbiome, but exactly how the genes influence changes in the gut microbiome requires further investigations. Further studies are also warranted to decipher how changes in the gut microbiome leads to the development of autoimmunity and T1D.

## 5 Discussion

In this perspective article, we delve into the multifaceted relationship between genetic predispositions and environmental factors in the onset and progression of T1D. This exploration is crucial, as it provides insights into how specific genetic profiles interact with environmental triggers, leading to the development of T1D.

Although pathophysiology of T1D is complex, genetics has been strongly implicated in the development of disease. Gene variants in *INS*, *GLIS3*, *CCR5*, *BAD*, *GPX7*, *GSTT1*, and *SNX19* have been associated with T1D (9–16) (**Table 1**). However, not all the individuals harboring these gene variants develop T1D again highlighting the crucial role of gene-environment interplay in predisposition to T1D.

Besides genetics, environmental factors such as viral infections and pesticide exposure have been implicated with the development of T1D (18–22) (**Supplementary Table 1**). However, the causal relationship between viral infections and T1D remains complex and multifaceted. Not all individuals exposed to diabetogenic viruses develop T1D. Although compelling evidence supports the association between viral infections and T1D, further research is warranted regarding the specific viruses involved, timing of infection, the underlying molecular mechanisms of immune dysregulation, and the potential for preventive interventions. In a similar context, while pesticide exposure is being investigated as a potential environmental risk factor for T1D, there is a need to understand its interaction with genetic susceptibility, viral infections, and other environmental influences. This comprehensive understanding is vital for unraveling the complex etiology of the disease. A better knowledge about the causative relationship between pesticide exposure and T1D can contribute to preventive strategies and public health recommendations. Individuals, especially those in occupations with potential pesticide exposure and families residing in agricultural areas, should be aware of potential risks and take appropriate precautions to minimize exposure. Additionally, ongoing surveillance and research are crucial to further elucidate the impact of pesticide exposure on T1D.

Although the precise molecular mechanisms through which gene-environment dynamic interplay leads to the development of T1D are still not clear, epigenetics and changes in gut microbiome have been hypothesized to play a pivotal role. Epigenetic changes such as abnormal methylation patterns can occur in genes related to immune function or insulin production, altering their expression, and potentially triggering an autoimmune response against pancreatic beta cells (37, 61–66). While there has been significant progress in understanding how epigenetics contributes to T1D, several research gaps remain that need to be addressed for a more comprehensive understanding. There is a need to understand the causal relationship between epigenetic modifications and the initiation and progression of T1D. Deciphering the role of epigenetics will provide a deeper understanding of T1D etiology.

The changes in gut microbiome have been hypothesized to play a pivotal role in gene-environment interaction and development of T1D (77–81). Although some progress has been made in understanding the role of the gut microbiome in T1D, there are still many unanswered questions. The exact mechanisms by which alterations in the gut microbiota leads to autoimmune responses against pancreatic β-cells are not fully understood. Understanding these mechanisms is crucial for developing potential therapeutic interventions. Furthermore, there is a need to perform more longitudinal studies to understand how early-life exposures and changes in the gut microbiome contribute to the development of T1D, especially using emerging techniques such as omics technology. The information derived from these studies would provide insights into the temporal dynamics of microbiome changes and their association with T1D onset.

## 6 Conclusions and future directions

The availability of preclinical animal models and epidemiological studies using large cohorts can significantly increase our understanding regarding the role of gene-environment interplay in the molecular underpinnings of T1D. There is a need to discover novel therapeutic interventions that facilitate demethylating key DNA regions implicated in the pathogenesis of T1D. In addition, there is a need to understand the precise functional role of the gut microbiome in the pathophysiology of T1D using emerging omics technologies. Simultaneously, there is a critical emphasis on developing therapeutic strategies aimed at reducing gut dysbiosis observed in T1D individuals and restoring the normal gut microbiome. Considering the crucial role of epigenetics in the disease process, the other avenue of research should be focused on determining the efficacy of “epidrugs” already available in the market for prevention and treatment of T1D (84). Repurposing Food and Drug Administration (FDA) approved drugs significantly reduces the cost, time, labor, and high-risk process of drug development with greater rates of success. The development of novel interventions that focus on the interplay between genes and the environment offers significant hope for the efficient management of T1D in pursuit of improving the quality of life of affected individuals and their families/caregivers.

## Data availability statement

The original contributions presented in the study are included in the article/**Supplementary Material**. Further inquiries can be directed to the corresponding authors.

## Author contributions

RM: Conceptualization, Data curation, Formal analysis, Investigation, Methodology, Project administration, Resources, Supervision, Validation, Visualization, Writing – original draft, Writing – review & editing. NC: Conceptualization, Investigation, Methodology, Validation, Visualization, Writing – original draft, Writing – review & editing. JL: Conceptualization, Formal analysis, Investigation, Methodology, Project administration, Supervision, Validation, Visualization, Writing – original draft, Writing – review & editing. KH: Conceptualization, Data curation, Formal analysis, Investigation, Methodology, Project administration, Supervision, Validation, Visualization, Writing – original draft, Writing – review & editing.
