# Supplementary material for: Gene-environment interaction in the pathophysiology of type 1 diabetes
Source: Front Endocrinol (Lausanne). 2024 Jan 26;15:1335435. doi: 10.3389/fendo.2024.1335435 (PMC10858453; doi:10.3389/fendo.2024.1335435)
Supplement: Supplementary file 1 [file Table_1.pdf]

Supplementary Table 1: Environmental factors involved in the pathophysiology of T1D.

Environmental Factor	Potential Molecular Mechanisms	References
Viral Infections	Certain viruses such as enteroviruses are associated with triggering T1D, possibly by causing an autoimmune response against pancreatic cells.	(20-22)
Pesticide Exposure	Pesticides may trigger or accelerate the autoimmune response that leads to beta-cell destruction in the pancreas.	(39, 40)
Mode of delivery and antibiotic use	May influence the gut microbiota development leading to immune dysregulation.	(41-43)
Lifestyle and Dietary Factors	Early exposure to cow's milk, short breastfeeding duration, and early introduction of cereals and gluten might increase risk by immune dysregulation and modulating gut microbiota.	(43)
Vitamin D Deficiency	Lower levels of Vitamin D are observed in individuals with T1D, suggesting a potential role in disease development through immune modulation.	(47-50)
